# Sex hormone levels in females of different ages suffering from depression

**DOI:** 10.1186/s12905-021-01350-0

**Published:** 2021-05-22

**Authors:** Rong Lei, Yan Sun, Jiawen Liao, Yuan Yuan, Linlin Sun, Yugeng Liu, Xinyu Yang, Wenyou Ma, Zhenjian Yu

**Affiliations:** 1 Department of Adult Psychiatry, Second People’s Hospital of Huizhou, Huizhou, China; 2 Department of Clinical Psychology, Kailuan Mental Health Centre Affiliated to North China University of Science and Technology, Tangshan, China; 3 School of Psychology and Mental Health, North China University of Science and Technology, Tangshan, China; 4 Department of Geriatric Psychiatry, Second People’s Hospital of Huizhou, Huizhou, China; 5 School of Basic Medical Sciences, Peking University Health Science Center, Beijing, China; 6 Department of Mood Disorders, Tianjin Mental Health Centre, Tianjin, China; 7 Department of Clinical Psychology, Kailuan Mental Health Center Affiliated to North China University of Science and Technology, Tangshan, China

**Keywords:** Sex hormones, Female, Depression, Age, Estradiol

## Abstract

**Background:**

There are only a few studies on sex hormones in females of different ages suffering from depression, and their conclusions are not uniform until now. This study aimed to investigate the correlation between the severity of depression in females and factors such as sex hormones and differences in sex hormone levels in females of different ages, exploring variations after treatment.

**Methods:**

A total of 169 females with depression were selected and divided into the first-episode (91 cases) and recurrent (78 cases) groups. Then, on the basis of their age, the first-episode patients were divided into the young (48 cases, age < 45 years), perimenopausal (20 cases, 45–55 years), and elderly groups (23 cases, age > 55 years); the patients with recurrent depression were classified into the young (37 cases, age < 45 years), perimenopausal (19 cases, 45–55 years), and elderly groups (22 cases, age > 55 years). The patients were assessed in accordance with the *International Classification of Diseases* of mental and behavioral disorders. The serum progesterone, prolactin, estradiol, and testosterone levels in the patients were measured, and differences in sex hormone levels of the groups were analyzed.

**Results:**

The estradiol level was negatively correlated with age and the prolactin level was positively correlated with occupation. The severity of depression in females was found to be negatively correlated with age. The serum progesterone and estradiol levels in the young group were significantly higher than those in the elderly group, regardless of the first episode or recurrence. Estradiol levels in the perimenopausal and elderly groups with first-episode depression were significantly higher than those in the same group with recurrent depression. However, there was no significant difference in the serum progesterone, prolactin, estradiol, and testosterone levels in the recurrent group before and after treatment.

**Conclusions:**

Sex hormone levels, especially estradiol, varied among females of different ages suffering from depression. Recurrent depression also has a certain effect on sex hormone levels in females. Not only should the age and relapse be considered when studying the sex hormone levels of females with depression, but also attention should be paid to whether the patients have used antidepressants before their sexual hormonal testing.

## Background

Nowadays, depression has become one of the most common mental disorders and is regarded as the most severe disability/disease in the world [1], accounting for one-third of the social and economic cost of the global burden of disease. It is also expected to become the most socioeconomically burdened disease in the world by 2030 [2], leading to a serious threat to both developed and developing countries. Its diagnosis is broad and heterogeneous that centers on low mood and/or loss of pleasure in activities. The induction of depression may be related to biology, psychology, social environment, and other factors. However, the specific mechanism is unclear. Depression appears to be more common in women than in men, with women having twice the risk as that of men. The recurrence is an important factor in determining that women are more likely to develop active depression compared to men once the first episode has occurred, but the reasons for this gender difference are not entirely clear [3]. Before puberty, girls were found to have depression levels similar to those in boys or lower [4]. Two female sex hormones—estrogen and progesterone—are considered to play a role in the onset and development of depression [5].

Studies have found that sex hormones have dramatic effects on females’ lives, and anxiety-, trauma-, and stress-related disorders are modulated by sex hormones [6, 7]. Nearly all women experience hormonal fluctuations associated with menstruation, pregnancy, and menopause, and some even undergo severe premenstrual [8] and perimenopausal/postpartum depression [9]. These women experience typical changes of reproductive hormone and suboptimal responses of the central nervous system, leading to negative effects and incommensurate behaviors [10]. Postmenopausal endocrinology is characterized by persistent low levels of ovarian steroid hormones (estradiol and progesterone) and a 50 % decrease in the secretion of testosterone compared to young women. Therefore, women are more likely to develop depression at the beginning or after menopause, the severity of which is linked to fluctuations in ovarian hormone levels—particularly estrogen [11]. Moreover, studies have demonstrated that estrogen has a positive influence on the mood of middle-aged women [12]. Of note, the revised Global Consensus Statement on Menopausal Hormonal Therapy suggests that hormone therapy may be conducive to improving mood among women in early postmenopause with symptoms of depression/anxiety [13]. Estrogen increases the synthesis and effectiveness of serotonin by reducing the activity of the serotonin-degrading enzyme monoamine oxidase inhibitor [3]. Serotonin is released into the area that regulates the amygdala’s response, and involved in mood regulation. Steroid hormones, also known as neuroactive steroids, are involved in emotional and cognitive regulation through their organization and activation in the central nervous system and may be effective treatment strategies for schizophrenia and depression [3]. It is worth noting that the peripartum is also a period of dramatic changes in hormone levels. Prolactin, which is responsible for initiating and maintaining lactation, is used as one of the measurement indicators to estimate the relationship between mood and hormonal changes in postpartum women. Estrogen can stimulate the secretion of prolactin, the level of which (similar to estrogen) decreases rapidly after parturition [14]. The degree of depression is the ultimate manifestation of the interaction of these hormonal variables. As the concentration of sex hormones continues to change throughout a female’s life cycle, changes in hormone levels can affect the response to antidepressant medications and the occurrence of adverse events. The induction/inhibition of estrogen and progesterone enzymes may lead to an increase/decrease in the metabolism of antidepressants during pregnancy. Thus, postmenopausal women (with decreased estradiol and progesterone levels and increased luteinizing hormone levels) may require different treatments/antidepressant dosages compared to premenopausal ones.

The sex hormone levels are affected by many factors, including age and health status, among which age are considered the main factor [15, 16]. At the ovarian level, the number of follicles and their viability and secretions of estrogen, progesterone, inhibin, and activin fluctuate with age. However, at present, research on sex hormones in female patients of different ages suffering from depression is still relatively rare, and the conclusions are not uniform. Hence, this study aimed to compare the sex hormone levels in females of different ages suffering from depression and provide clinical references for future research on the characteristics of these hormone levels in such females.

## Methods

### Patients

This study was approved by the Ethics Committee of Kailuan Mental Health Center Affiliated to North China University of Science and Technology, Hebei, China. A total of 169 females with depression were selected between September 2017 and March 2019 at Kailuan Mental Health Center Affiliated to North China University of Science and Technology. The chief psychiatrist made the diagnosis for patients with depression according to the *International Classification of Diseases* (ICD-10) of mental and behavioral disorders. Patients with no previous history of depressive episodes were classified as the first-episode group. Those with recurrent depressive disorder were preliminarily diagnosed using the above criteria, and finally a definitive diagnosis was made by a superior psychiatrist. The patients were divided into the first-episode (91 cases) and recurrent (78 cases) groups. Then, the first-episode patients were divided into the young (48 cases, age < 45 years), perimenopausal (20 cases, 45–55 years), and elderly groups (23 cases, age > 55 years) on the basis of their age. Similarly, the patients with recurrent depression were classified into the young (37 cases, age < 45 years), perimenopausal (19 cases, 45–55 years), and elderly groups (22 cases, age > 55 years) according to their age.

#### Inclusion criteria

The patients met the diagnostic criteria for depression according to the ICD-10 classification of mental and behavioral disorders.

#### Exclusion criteria

(1) failure to meet the inclusion criteria; (2) current/previous neurological disorders, brain trauma, bipolar disorder, manic episodes, substance abuse (methamphetamine, cocaine, etc.), and other mental illnesses; (3) presence of severe cerebrovascular and brain diseases, such as cerebral infarction and hemorrhage; (4) women using hormones/contraceptives; (5) gynecological, immunological, endocrine, and other diseases; (6) people who dropped out or failed to complete the investigation because of other factors.

### Diagnostic assessment

(1) *Depressive episode* typical symptoms of depression including low mood, loss of interest and pleasure, fatigue, and other common symptoms: (a) deterioration of ability to concentrate; (b) low self-evaluation and confidence; (c) sense of self-incrimination and worthlessness; (d) pessimism about the future; (e) autotomy/suicide idea or behavior; (f) sleep disorder; (g) loss of appetite. These symptoms persist for at least 2 weeks.

Mild depressive episode: At least two mild typical symptoms of depression and other common symptoms, lasting for at least 2 weeks. Moderate depressive episode: at least two mild typical symptoms of depression and three mild common symptoms (preferably four), lasting at least 2 weeks. Major depressive episode (without psychotic symptoms): three typical symptoms of depression and four or more common symptoms should generally last for 2 weeks, but it is reasonable to make this diagnosis on the basis of a course of less than 2 weeks when symptoms are extremely severe or onset is rapid. Major depressive episode (with psychiatric symptoms): meet the criteria for major depressive episodes and have delusions, hallucinations, or depressive stupor. The severity of symptoms is assessed on the basis of difficulty in completing daily tasks and social interactions, such as decreased interest/pleasure, weight and appetite changes, sleep disturbance, and significant distress or impairment in social, occupational, or other areas.

(2) *Recurrent depressive disorder* recurrent depression (mild/moderate/major) without independent episodes of hyperthymia and hyperactivity that meet manic criteria.

Mild recurrent depressive episode: meet the criteria for mild recurrent depressive disorder, at least two episodes, each lasting for at least 2 weeks, with no apparent mood disorder between the episodes for several months. Moderate recurrent depressive episode: meet the criteria for moderate recurrent depressive disorder, at least two episodes, each lasting for at least 2 weeks, with no apparent mood disorder between the episodes for several months. Major recurrent depressive episode (with/without psychiatric symptoms): meet the criteria for severe recurrent depressive disorder with/without psychiatric symptoms, at least two episodes, each lasting for at least 2 weeks, with no apparent mood disorder between the episodes for several months.

### Treatments

Patients with recurrent depressive disorder were treated with duloxetine at 40–60 mg/d for 3 months until the end of the second sex hormone test. During this period, appropriate benzodiazepines and other drugs were used for adjuvant therapy depending on the patients’ conditions.

### Parameters

General information about all the patients was collected, including their gender, age, height, weight, education level, occupation (workers, farmers, cadres, retired, none, and other), smoking and drinking habits (judgment standard of history of smoking: smoking more than one cigarette a day, continuous/cumulative 6 months; history of drinking criterion: more than 500 g/350 g [male/female] per week and persisting for longer than 5 years), complications (mainly for high blood pressure, diabetes, coronary heart disease, stroke, none, and others; the judgment criterion is based on the diagnosis of the corresponding disease or the usage of related drugs), marital status, and records of the patients’ use of antidepressants in the last week.

### Detection of sex hormone levels

Outpatients were tested for sex hormone levels on the same day. Inpatients with recurrent depressive disorder underwent the first assessment of sex hormone levels within 24 h of admission and the second assessment of sex hormone levels after 3 months of treatment. The5 mL of cubital venous blood between 8 and 10 am was taken on an empty stomach. The magnetic particle-based chemiluminescence method was used to detect samples using a chemiluminescence detector (AutoLumo A200) according to the instructions of the adrenocorticotropic hormone detection kit (ACTH CLIA Microparticles).

### Statistical analysis

The SPSS (v.22.0; IBM Corp., Armonk, NY, USA) was used to analyze the data collected. A chi-squared test or Fisher’s exact probability was performed to compare the count data between the two groups. Pearson’s correlation analysis was applied to analyze the correlation between sex hormone levels and age and body mass index (BMI). Spearman’s correlation analysis was used to estimate the correlation between sex hormone levels and classification variables such as occupation, as well as the correlation between the severity of depression and related factors. Sex hormone levels in different age groups were compared by one-way analysis of variance, followed by Bonferroni’s correction. Sex hormone levels were compared between the first-episode and recurrent groups using independent *t*-tests. The comparison of sex hormones in the recurrent group between pre- and posttreatment was performed by a paired-sample *t*-test with the test standard *α* = 0.05.

## Results

### Case data

The clinical data of the included cases are shown in Table 1. A total of 169 subjects were included in the study. The average age of the first-episode group was 42.98 ± 14.84 years, ranging from 16 to 83 years. That of the recurrent group was 48.02 ± 12.59 years, with the youngest being 27 years old and the oldest 76 years old. There were no significant differences in age, smoking and drinking habits, and BMI (*P* > 0.05) between the first-episode and recurrent groups. Differences in marital status, occupation, and complications between the first-episode and recurrent groups were observed (*P* < 0.05).

**Table 1:** Clinical data of the cases

Variables		Cases		*χ*^2^	*P*
		First-Episode	Recurrence		
Age				0.905	0.636
	< 45	48	37		
	45–55	23	19		
	> 55	20	22		
Smoking and drinking habit					
	Yes	0	3	-	0.096
	No	91	75		
Marital status				12.880	0.001*
	Married	81	72		
	Unmarried	9	0		
	Divorce	0	2		
	Remarriage	1	3		
	Widowed	0	1		
Occupation				26.422	0.000*
	Worker	20	32		
	Farmer	10	3		
	Cadre	6	10		
	Retirement	15	6		
	Student	15	11		
	Staff	11	16		
	None	7	0		
	Other	7	0		
Complication				8.797	0.015*
	Hypertension	1	0		
	Diabetes	1	2		
	Cardiopathy	0	1		
	None	89	68		
	Other	0	4		
	Multi-symptom				
BMI(kg/m^2^)		23.48 ± 2.68	24.24 ± 3.54	− 1.595	0.113
Antidepressant use				66.97	0.000*
	Yes	22	68		
	No	69	10		

The statistical software did not give a corresponding value (-); Fisher’s exact probability was performed (*). BMI: body mass index

### Correlation analysis of sex hormones and sociodemographic variables

This correlation analysis (Table 2) showed that the estradiol level was negatively correlated with age (*r* = − 0.267, *P* = 0.000) and the prolactin level was positively correlated with occupation (*r* = 0.157, *P* = 0.041).

**Table 2:** The correlation between sex hormones and sociodemographic variables

Sex hormones	Age	BMI (kg/m^2^)	Occupation	Smoking and drinking habit	Complication
P	− 0.087	0.018	0.008	− 0.005	0.044
PRL	− 0.009	− 0.027	0.157^b^	− 0.105	− 0.039
E_2_	− 0.267^a^	− 0.055	0.119	0.055	0.057
T	− 0.053	− 0.094	0.100	0.042	− 0.041

a, *p* = 0.000; b, *P* = 0.041. P: progesterone; PRL: prolactin; E2: estradiol; T: testosterone; BMI: body mass index

### Correlation analysis of the degree of depression with age and sex hormones in females

The severity of depression in females was negatively correlated with age (*r* = − 0.205, *P* = 0.007), but it exhibited no significant correlation with occupation, smoking and drinking habits, comorbidities, and serum progesterone, prolactin, estradiol, and testosterone levels (all *P* > 0.05; Table 3).

**Table 3:** The correlation between the degree of depression and related factors in females

Items	Age	Job	Smoking and drinking habit	Complication	*P*	PRL	E_2_	T
Severity	− 0.205	− 0.005	0.044	0.056	0.084	0.021	0.103	0.097
*P*	0.007	0.945	0.561	0.464	0.275	0.784	0.181	0.210

P: progesterone; PRL: prolactin; E2: estradiol; T: testosterone

### Sex hormone levels in females with depression in different age groups

As shown in Table 4, in the first-episode group, the serum progesterone level in the young group of females with depression was significantly higher than that in the elderly group (*P* < 0.05); There was no significant difference in prolactin levels among the young, perimenopausal, and elderly groups (*P* > 0.05); The estradiol level in the young group was significantly upregulated compared to the elderly group (*P* < 0.05), and the estradiol level in the perimenopausal group was higher than that in the elderly group (*P* > 0.05); The testosterone level in the young and elderly groups was higher than that in the perimenopausal group, whereas there was no significant difference among the three groups (*P* > 0.05).

**Table 4:** Sex hormone levels in females of different ages suffering from depression

Variables	Young group	Perimenopausal group	elderly group	*P1*	*P2*	*P3*
First-Episode	n = 48	n = 23	n = 20			
P (ng·mL^− 1^)	3.17 ± 5.03	1.35 ± 2.51	0.41 ± 0.29	0.064	0.010	0.429
PRL (uIU· mL^− 1^)	685.58 ± 1153.46	630.23 ± 1308.88	690.35 ± 972.45	0.851	0.988	0.867
E_2_ (pg·mL^− 1^)	77.19 ± 72.48	68.68 ± 70.81	33.05 ± 18.43	0.602	0.014	0.076
T (ng·mL^− 1^)	0.76 ± 2.42	0.37 ± 0.13	0.51 ± 0.77	0.399	0.611	0.810
The-Recurrence	n = 37	n = 19	n = 22			
P (ng·mL^− 1^)	3.93 ± 7.94	2.19 ± 4.49	0.54 ± 0.28	0.303	0.037	0.376
PRL (uIU· mL^− 1^)	392.28 ± 357.01	697.27 ± 770.34	530.56 ± 633.86	0.058	0.364	0.347
E_2_ (pg·mL^− 1^)	81.12 ± 77.70	33.84 ± 25.09	19.77 ± 11.20	0.003	0.000	0.421
T (ng·mL^− 1^)	0.98 ± 1.48	0.71 ± 1.54	0.44 ± 0.77	0.480	0.133	0.509

*P1*: comparison between the young group and the perimenopausal group; *P2*: comparison between young group and elderly group; *P3*: comparison between perimenopausal group and elderly group. P: progesterone; PRL: prolactin; E2: estradiol; T: testosterone

In the recurrent group. the serum progesterone level in the young group was significantly elevated in contrast to the elderly group (*P* < 0.05), and the serum progesterone level in the perimenopausal group was higher than that in the elderly group (*P* > 0.05); No significant difference was observed in prolactin and testosterone levels among the young, perimenopausal, and elderly groups (*P* > 0.05); The estradiol level in the young group was significantly higher than that in the perimenopausal and elderly groups (*P* < 0.05), and the estradiol level in the recurrent perimenopausal group was higher than that in the elderly group (*P* > 0.05).

### Comparison of sex hormone levels in females with first-episode and recurrent depression

The estradiol level in the first-episode perimenopausal group was obviously enhanced compared to the recurrent perimenopausal group (*P* < 0.05; Table 5). The estradiol level in females with depression in the first-episode elderly group was significantly higher than that in the recurrent elderly group (*P* < 0.05; Table 5). There was no significant difference in other sex hormone levels between the first-episode and recurrent groups (all *P* > 0.05; Table 5).

**Table 5:** Comparison of sex hormone levels in females with first-episode and recurrent depression

Variables	First-Episode	Recurrence	*t*	*P*
*Young group*	n = 48	n = 37		
P (ng·mL^− 1^)	3.15 ± 5.10	3.98 ± 7.73	− 0.581	0.563
PRL (uIU· mL^− 1^)	618.54 ± 1147.57	388.41 ± 347.90	1.204	0.232
E_2_ (pg·mL^− 1^)	75.83 ± 74.00	80.05 ± 75.77	− 0.255	0.799
T (ng·mL^− 1^)	0.79 ± 2.51	0.98 ± 1.48	− 0.419	0.677
*Perimenopausal group*	n = 27	n = 19		
P (ng·mL^− 1^)	1.35 ± 2.51	2.10 ± 4.39	− 0.710	0.482
PRL (uIU· mL^− 1^)	630.23 ± 1308.88	697.27 ± 770.34	− 0.198	0.844
E_2_ (pg·mL^− 1^)	68.68 ± 70.81	33.38 ± 24.51	2.284	0.030
T (ng·mL^− 1^)	0.37 ± 0.13	0.69 ± 1.50	− 0.930	0.364
*Elderly group*	n = 19	n = 22		
P (ng·mL^− 1^)	0.41 ± 0.29	0.54 ± 0.28	− 1.465	0.151
PRL (uIU· mL^− 1^)	690.35 ± 972.45	530.56 ± 633.86	0.631	0.531
E_2_ (pg·mL^− 1^)	33.05 ± 18.43	19.77 ± 11.20	2.832	0.007
T (ng·mL^− 1^)	0.51 ± 0.77	0.44 ± 0.77	0.291	0.772

P: progesterone; PRL: prolactin; E2: estradiol; T: testosterone

### Comparison of sex hormone levels in the recurrent group before and after treatment

After three months of treatment, the serum sex hormone level in the recurrent groups of all ages changed, but there was no significant difference (*P* > 0.05; Table 6). It was observed that treatment led to an increase in the progesterone level in the young group and a reduced progesterone concentration in the perimenopausal group. After treatment, prolactin levels increased in the young and elderly groups and decreased in the perimenopausal group. Estradiol levels decreased in the young group and increased in the perimenopausal and elderly groups after the patients received treatment. No overt alteration was observed in testosterone levels among the three groups before and after treatment.

**Table 6:** Comparison of sex hormone levels in the recurrent group before and after treatment

	Variables	Prior treatment	Post-treatment	*t*	*P*
Young group (n = 37)	P (ng·mL^− 1^)	3.98 ± 7.73	5.29 ± 7.99	− 0.764	0.449
	PRL (uIU· mL^− 1^)	388.41 ± 347.90	590.48 ± 1257.69	− 0.987	0.330
	E_2_ (pg·mL^− 1^)	80.05 ± 75.77	66.76 ± 64.80	0.924	0.362
	T (ng·mL^− 1^)	0.98 ± 1.48	1.15 ± 1.83	− 0.671	0.507
Perimenopausal group (n = 19)	P (ng·mL^− 1^)	1.43 ± 3.29	0.55 ± 0.28	1.152	0.265
	PRL (uIU· mL^− 1^)	668.58 ± 786.31	371.45 ± 245.61	1.632	0.120
	E_2_ (pg·mL^− 1^)	30.10 ± 20.19	35.21 ± 32.43	− 0.749	0.463
	T (ng·mL^− 1^)	0.72 ± 1.54	0.63 ± 1.13	0.192	0.850
Elderly group (n = 22)	P (ng·mL^− 1^)	0.54 ± 0.28	0.57 ± 0.30	− 0.616	0.544
	PRL (uIU· mL^− 1^)	530.56 ± 633.86	637.63 ± 868.49	− 0.669	0.511
	E_2_ (pg·mL^− 1^)	19.77 ± 11.20	24.07 ± 11.75	− 1.155	0.261
	T (ng·mL^− 1^)	0.44 ± 0.77	0.35 ± 0.17	0.479	0.637

P: progesterone; PRL: prolactin; E2: estradiol; T: testosterone

## Discussion

Epidemiological data show that about 20 % of females suffer from major depression at some point during their lives. Notably, for some women, depression can appear or worsen in different periods, during which hormone levels dynamically change, including premenstrual, perinatal, and perimenopausal periods [17]. Mounting evidence has proved that sex hormones participate in the physiological and pathological development of depression in females [14, 18, 19]. Nevertheless, the relationship between depression and sex hormones in women remains to be further studied.

There are many factors that induce depression. For example, genetic factors—that is, congenital defects—may lead to weaker stress tolerance. Studies have confirmed that depression has a certain family genetic risk. However, the specific cause is not clear. Several studies have also shown that there is a link between sex hormones and depression [20]. Some studies have suggested that abnormal sex hormone levels may be a clinical phenomenon in those suffering from depression [21]. This study found that progesterone and estradiol levels were significantly different in the first episode, recurrence, and among different age groups. This indicates a close relationship between depression and sex hormones. The role of sex hormones in regulating females’ emotions has been confirmed by many studies [22, 23]. As steroid hormones, progesterone and estradiol play vital roles in regulating brain morphology and function. Fluctuations in sex hormone levels can cause changes in the neurotransmitter system, thereby potentially and lastingly affecting mood and behavior. Estradiol is known to have protective effects on the nerves, especially for perimenopausal and elderly women [24, 25]. It has been found that γ-aminobutyric acid (GABA) or glutamic acid can affect mood by regulating the activity of the lateral reins (LHb) in the brain region [26, 27], and sex hormones have been found to interact with GABA and brain-derived neurotrophic factors in a way that affects mood [28–32]. In addition, certain sex hormones—such as progesterone, which is a positive phase-change structural regulator of GABA receptors, and it has been found that progesterone levels increased after treatment with antidepressants [33]. However, some studies on perimenopausal depression in females show that perimenopausal depression has nothing to do with the fluctuation in sex hormone levels, which is caused by the difficulty in measuring sex hormones [34]. A lot of research has been conducted on hormone therapy for people with depression. The vast majority of studies suggest that a combination of hormone therapy or medication should be given to those who are eligible, a treatment that deserves the attention of psychiatrists [35]. Of course, abnormal sex hormone levels in patients with depression are not only manifested in females, but also in males [36, 37]. And various sex hormones have a certain impact on people’s mental health to some extent, especially the elderly[38]. Moreover, the severity of depression in females was found to be negatively correlated with age, suggesting that young women were more prone to depression [39]. The severity of depression in elderly women seems to have received a lot of attention [40]. However, studies have pointed out that the increase of depression in elderly women was more strongly related to physical diseases (such as chronic bronchitis and obstructive emphysema, hypertension, cerebrovascular disease, and cataract) than to age [41].

From the life course of female patients with depression, they have experienced from relatively high sex hormone levels when they were young, to relatively low sex hormone levels during perimenopausal period, and then to further low sex hormone levels in the old age, so it can be seen that the sex hormone levels of female patients with depression change with age. The results of this study showed that sex hormone levels in females with depression were negatively correlated with age. As age increases, these sex hormone levels decrease, especially estradiol. This study also found that the progesterone and estradiol levels in young females with depression were significantly higher than those in the elderly group, regardless of the first episode or recurrence. Changes in hormone levels with age were also seen in healthy females, but changes in females with depression were more dramatic than those in healthy females, and these fluctuations in sex hormone levels have the potential to induce negative emotions [42]. Related studies have found that sex hormones play different roles in women with depression in different age groups [43].

The study also noted differences in sex hormone levels of females with depression in the first episode and in those with recurrent depression. We found that in the perimenopausal group, females with the first episode exhibited higher estradiol levels than those with recurrence. We believe that this difference in sex hormone levels is mainly due to the use of antidepressants. After all, patients with recurrent depression actively take antidepressants when their symptoms appear and do not come to the hospital until the drugs are ineffective. Accordingly, antidepressants have been taken for a period prior to the detection of sex hormones. Relevant studies have shown that antidepressants can affect sex hormone levels [44, 45]. Hence, it is reasonable to believe that the difference in estradiol levels between females with the first episode and recurrence of depression was due to the use of antidepressants. Of note, it has been proved that duloxetine may have endocrine-disrupting effects on the synthesis of steroids [46, 47]. In view of this, we speculated that the changes in sex hormone levels were likely to be attributed to the regulatory role of duloxetine in the endocranium. However, it remains to be investigated whether all antidepressants can affect sex hormone levels and whether these antidepressants have similar effects.

There are some limitations to this study. It lacks healthy controls and is limited to the small sample size, which makes the conclusions of this study less convincing. We intend to expand the sample size and add hierarchical analysis in the in-depth study. Furthermore, considering that many factors are responsible for depression—including biological, psychological, social environment, and genetic factors—patients with depression triggered by different situations are divided into corresponding groups to eliminate the interference before the analysis. Future research can further explore recurrence factors on the influence of sex hormone levels in females with depression. In addition, the effects of drug use, severity, and accompanying symptoms of anxiety on sex hormones in patients with first episode and recurrence can also be studied to make the study more comprehensive.

## Conclusions

The severity of depression in females was negatively related to age; the estrogen level in women with depression was negatively related to age; recurrent depression also had a certain effect on the sex hormones of females with depression. These results laid a foundation for the mechanism of sex hormones in depression and brought good news for the prevention and treatment of depression.

## Data Availability

The datasets used and/or analyzed during the current study available from the corresponding author on reasonable request.

## Abbreviations

BMI: Body mass index
GABA: γ- aminobutyric acid
P: Progesterone
PRL: Prolactin
E2: Estradiol
T: Testosterone
